# Impact of Urbanization on PM_2.5_-Related Health and Economic Loss in China 338 Cities

**DOI:** 10.3390/ijerph17030990

**Published:** 2020-02-05

**Authors:** Beidi Diao, Lei Ding, Qiong Zhang, Junli Na, Jinhua Cheng

**Affiliations:** 1 School of Economics and Management, China University of Geosciences, 388 Lumo Road, Wuhan 430074, China; beididiao@cug.edu.cn; 2 Institute of Environmental Economics Research, Ningbo Polytechnic, 388 Lushan Road, Ningbo 315800, China; 3 School of Industrial and Commercial, Ningbo Polytechnic, 388 Lushan Road, Ningbo 315800, China; jean_009@163.com; 4 Arts Faculty, Monash University, Victoria State, Melbourne 3800, Australia; najunli8844@sina.com

**Keywords:** PM_2.5_ concentration, urbanization level, public health, relative risk, economic loss

## Abstract

According to the requirements of the Healthy China Program, reasonable assessment of residents’ health risks and economic loss caused by urban air pollution is of great significance for environmental health policy planning. Based on the data of PM_2.5_ concentration, population density, and urbanization level of 338 Chinese cities in the year of 2015, the epidemiological relative risk (RR) was adopted to estimate the negative health effects caused by exposure to PM_2.5_. Meanwhile, the Value of Statistical Life (VSL) and Cost of Illness (COI) methods were used to calculate economic loss. The results show that PM_2.5_ pollution remains serious in 2015, which brings about many people suffering from all kinds of fearful health problems especially premature death and related diseases. The mortality and morbidity increase dramatically, and the total direct economic loss related to PM_2.5_ pollution in 2015 was 1.846 trillion yuan, accounting for 2.73% of total annual GDP. In addition, there was a strong correlation between urbanization level and health risks as well as economic loss, which implies that people who live in highly urbanized cities may face more severe health and economic losses. Furthermore, 338 cities were divided into four categories based on urbanization level and economic loss, of which the key areas (type D) were the regions where an increase in monitoring and governance is most needed. In the process of urbanization, policy makers should pay more attention to health costs and regional differentiated management, as well as promote the construction of healthy cities more widely.

## 1. Introduction

Environmental public health policy and regional differentiated management are important contents and critical paths for the construction of Healthy China. Healthy China was launched as a public health program that provides a new guideline to implement the country’s Healthy China initiative and promote people’s health. In the process of rapid urbanization, the loss of public health and socioeconomic benefit induced by environment pollutions has received widespread attention from academia and society. The main topics of concern are the relationship between urbanization and air quality, the evaluation of impact on human health related to urban air pollution, and so on [1,2]. Therefore, in the context of new-type urbanization and the construction of the Healthy China program, it is particularly important to rationally evaluate and ensure the health of urban residents.

Previous studies generally supported the idea that the relationship between urbanization and residents’ health is complex [3,4,5]. Urbanization level is a comprehensive indicator that can reflect the level of urban development [6], therefore each dimension of urban life affects health in its own way and the effects vary with the specific social and cultural contexts [7,8]. On one hand, urban residents may benefit from improved living standards and health services; from this perspective higher levels of urbanization can reduce health risks [9]. On the other hand, if only taking the health problems induced by environmental pollution into consideration, especially the public health risks related to air pollution in developing country, there is no doubt that urbanization is linked to poorer health [10]. In addition short-term or long-term exposure to heavily polluted air (taking particulate matter such as PM_2.5_ as the main pollutant) can bring about damage to the human body’s systems of respiratory, cardiovascular, and immune, mutations in structure of DNA and chromosomes, and also has significant correlation with the premature death and birth defects of newborns [11,12,13]. The impact of air pollution on individual health is difficult to estimate. However, in the context of rapid urbanization, the high population density and mobility have led to a sharp increase in the number of people exposed to pollution [14,15], the effect in public health must be critical.

With the relevant public health research becoming more in-depth and detailed, the estimation of health loss caused by PM_2.5_ has attracted more attention. Following the emergence of plentiful results in the medical community, experts in the field of public health have proposed the concept of Global Burden of Disease (GBD), which utilizes a variety of tools including experiments, questionnaires, and field research to analyze how PM_2.5_ affects mortality, disease incidence, hospitalization rate, working hours, and so on [16,17,18]. The results show that if the annual average concentration of PM_2.5_ is reduced, the life expectancy of the population will increase [19] and the economic benefits will also been improved [20]. On the contrary, the increase of PM_2.5_ concentration will bring different degrees of damage and loss. For example, Li et al. evaluated the contamination hazard of 62 key environmental protection cities in China, showing that PM_2.5_ pollution caused about 125,000 premature deaths and economic loss of 570.5 billion yuan [21]. However, in the empirical study, due to the differences in urban selection, research methods, sample capacity, and other factors, the exposure response coefficient obtained from different epidemiological cases will be quite different, and will bring some deviation to the actual health and economic loss estimation results [22,23]. Studies in environmental economics have begun to spring up in recent years, and scholars have used the Value of Statistical Life (VSL), the Willingness to Pay (WTP), the Marginal Willingness to Pay (MWTP), and the Cost of Illness (COI) to assess the impact on the economy and calculate the amount of economic loss [24,25,26]. These studies provide useful ideas and an analytical framework for the scientific exploration on the comparison of air pollution related health loss and regional (at city level) differences.

On the whole, many existing studies focus on two aspects; one is the correlation relationship between urbanization and air pollution, the other is the assessment of health risks and economic loss caused by air pollution. However, there are still few studies that combine the two research directions for further study [3] to explore the relationship between urbanization and PM_2.5_ related health risks and economic loss. Compared with the concentration of air pollutants, health risks and economic loss are also affected by population density, per capita income, medical costs, and so on, all of which are strongly correlated with urbanization level.

From the above analysis, this paper provides a comprehensive health risks and economic loss assessment based on the PM_2.5_ concentration, urbanization, population density, and related medical data in 338 cities for the year of 2015. Then, health risks and economic loss under different urbanization levels are tested to explore their correlation with urbanization. Compared with the existing studies, the paper contributes to the literature in two ways. First, the health risks and economic loss of PM_2.5_ in China are evaluated on the city level, which is helpful for fully understand the huge threat brought by air pollution. As such, the assessment results can be used for economic valuation and decision making on the environmental health policy options. Second, this study focuses on the comparison of health risks and economic loss between different urbanization levels and cities. In terms of scientific significance, this study analyzes the impact of urbanization on public health related to PM_2.5_ based on the research of air pollution and urbanization and expands the depth of urbanization research. For the policy application, this study offers cost–benefit analysis and decision-making reference for the regional differentiation control, so as to reduce the loss of health benefits and offer the policy basis of air governance for the construction of Healthy China. This provides a rationale for strengthening the public health services and medical security, in order to make them more accessible for those at risk of PM_2.5_ exposure.

## 2. Materials and Methods

### 2.1. Data Sources

The data in this paper mainly involve five parts: air pollution concentration, population density, urbanization level, per capita Gross Domestic Product (GDP) and other related economic data, and outpatient services or hospitalization expenses and other medical related data.

In order to avoid the bias and data deficiency caused by using ground monitoring data and improve the accuracy of calculation results, we use PM_2.5_ concentration data based on satellite remote sensing [27,28]. In general, the data can effectively measure the average level of air pollution in a certain region [29]. Therefore, we adopt the global annual satellite-derived PM_2.5_ product in the year of 2015 provided by Atmospheric Composition Analysis Group from Dalhousie University to measure the PM_2.5_ pollution condition in Chinese cities [30].

From existing studies, we can make out that the end-of-year resident population is always used for assessment of health risk [31]. This population data ignores a large number of floating population and results in the final estimated results being biased. Therefore, this paper uses LandScan™ dataset [32] to estimate the population that are exposed to PM_2.5_ pollution more accurately. Specifically, the population scale of each city is calculated by LandScan™ dataset in the year of 2015 provided by Geographic Information Science and Technology (GIST) from Texas A&M University. In addition, per capita GDP and per capita disposable income data for the year of 2015 are from the China City Statistical Yearbook and the statistical yearbooks of each province in 2016. The unit outpatient service or hospitalization expenses and length of hospital stays is from the China Health and Family Planning Statistical Yearbook in 2016.

For the evaluation indicators of urbanization level, most existing studies use population urbanization rate to simply represent the level of urban development [33]. However, some scholars found that population urbanization rate is not powerful enough to express the level of multi-dimensional urban development [34]. Another reason is that China’s special household registration system makes the official urbanization rate quite different from the real urbanization level [35]. Elvidge et al. were the first to link nighttime light data to urban development [36]. After that, many studies also proved the rationality of using nighttime light data to measure the level of urban development [37]. The nighttime light data can distinguish urban regions from rural areas and reflects the basic information of human activities at night comprehensively, thus it can be used to represent urbanization level [38]. Visible Infrared Imaging Radiometer Suite Day (VIIRS) /Night Band Nighttime Lights annual data in 2015 from National Oceanic and Atmospheric Administration (NOAA) were selected to represent the indicator of urban development level. In the annual composites, the digital number (DN) value of each pixel is the average of the visible-band DN values of lights from cities, which is used to indicate the level of urbanization. In order to obtain the indicator of urbanization at city level, first Environment for Visualizing Images (ENVI) is used to eliminate temporary data and outliers as well as remove background noise [39,40]. Then zonal statistics in ArcGIS (Geographic Information Science) is used for urban average value extraction.

### 2.2. Methodology

#### 2.2.1. Health Risks Assessment

When it comes to the assessment of health risks due to air pollution, previous studies have generally obtained the exposure–response relationship between the concentration of air pollutants and the health effects. Then, based on the Poisson regression relative risk model, the changes of health effects caused by air pollution can be estimated [41,42].

In this model, the health risk (morbidity or mortality) related to PM_2.5_ pollution is set as:

(1) HI=[(RR−1)/RR]×BIR×EP

where HI represents the change in the health outcomes caused by PM_2.5_; EP is the exposed population; BIR is the baseline disease-specific health impacts rate; and [(RR − 1)/RR] is defined as the incidence of morbidity or mortality when an entire population be exposed to pollution. RR indicates relative risk factors; three RR estimation functions were developed for ambient PM_2.5_ exposure, including integrated exposure risk (IER) function, log-linear (LL) function, and non-linear power law (NLP) function. From the PM_2.5_-related mortality assessment studies, the IER has mostly been used [12], IER function can be expressed as follows:

(2) $${\mathrm{RR}}_{IER}=1+\alpha \left[1-\exp\left(-\beta {\left(c-c_{0}\right)}^{\delta }\right)\right]$$

For PM_2.5_-attributed morbidity studies, only LL has been used in the past literature [43], LL function can be calculated as follows:

(3) $${\mathrm{RR}}_{LL}=\exp\left[\gamma \times \left(c-c_{0}\right)\right]$$

where c represents the actual PM_2.5_ concentration; $c_{0}$ refers to the threshold concentration; α, β, δ determine the overall shape of the exposure–response relationship in IER; and γ is the exposure–response coefficient in LL.

However, there is a big difference in the selection of $c_{0}$ [42]. In the Air Quality Guidelines issued by the World Health Organization in 2005, the average annual exposure concentration of 10 μg/m^3^ is used as the guideline value for long-term exposure of PM_2.5_ [29]. This concentration is the lowest limit of the concentration observed to have a significant impact on survival rates in studies conducted by the American Cancer Society (ACS) [44]. Compared with the “Environmental Air Quality Standard” (GB3095-2012) issued by China Environmental Protection Agency (PM_2.5_), the reference value 10 μg/m^3^ in this paper is more stringent than that of the primary standard (15 μg/m^3^) and the second level standard (35 μg/m^3^). Moreover, the final estimated results of health risk related to PM_2.5_ pollution will also be larger and more serious. This will help policy makers reassess the health risks and serious challenges posed by PM_2.5_ pollution at a higher standard.

#### 2.2.2. Relative Risk and Baseline Incidence Rate

Exposure–response function analysis derives from a series of epidemiological studies about the effects of long-term exposure to polluted air on human health, such as the widely adopted research coefficient from studies of Harvard six cities and the American Cancer Society [44,45,46]. However, both of these studies were conducted against the background of low PM_2.5_ concentration in the United States, and the exposure–response coefficients obtained from these studies are not applicable to the current situation in China. Therefore, this paper refers to the studies of Maji et al. [12,47,48,49] on China, using their research results to assess the effects on different health outcomes. The specific coefficients used in this study are presented in Table 1.

#### 2.2.3. Economic Loss Estimate

There are two main kinds of economic loss caused by PM_2.5_ concentration. One is the economic loss caused by the premature death, which leads to the loss of labor force; the Value of Statistical Life (VSL) method is used for this estimation [50]. VSL does not mean the specific value of one person’s life, but rather in the statistical sense, the cost that people are willing to pay in order to reduce a unit risk of death, then measured in monetary terms [51]. The other one is the medical costs caused by the increase of the incidence of related diseases, which is measured by the Cost of Illness (COI) method [52]. The estimation methods are shown as follows:

From Aldy and Viscusi [53], the unit economic loss caused by the premature death (which is 1.68 million yuan in Beijing) and the ratio of disposable income are used for the calculation. The specific estimation formula is:

(4) $$\mathrm{DED}=\sum_{i}^{}1.68\times \frac{CDI_{i}}{CDI_{B}}\times EM_{i}$$

where DED represents the direct economic loss caused by the premature death; i means different areas; $CDI_{i}$ refers to per capita disposable income in i city; $CDI_{B}$ refers to per capita disposable income in Beijing; and $EM_{i}$ stands for mortality related to PM_2.5_ in i city.

Chronic bronchitis has a long course and it is not easy to be cured, so the time lost due to illness is difficult to determine. As chronic bronchitis often causes great pain to patients and reduces the quality of life of patients significantly, it is appropriate to use VSL method to calculate unit cost. Consequently, the research results that the unit cost of chronic bronchitis being 40% of the statistical life value from Viscusi and Cheng et al. are adopted here [54,55].

The loss of hospital admission and disease can be calculated by the Cost of Illness (COI), the loss of hospitalization include the hospitalization costs and the cost of missed work, the average daily value of per capita GDP can be regarded as the cost of daily missed work, and the time of missed work is the length of hospital admission. The unit economic loss of acute bronchitis and asthma is estimated by the research results of Huang and Zhang [26], combined with the loss of disease treatment in each province and the per capita disposable income of each city.

The specific estimation formulas are as follows [27]:

(5) $$\mathrm{DEH}=\sum_{ij}^{}HE_{ij}=\sum_{ij}^{}E_{ij}\times RP_{ij}+\frac{t_{i}}{250}\times GDP_{i}$$

(6) $$\mathrm{DEI}=\sum_{ij}^{}HE_{ij}=\sum_{ij}^{}E_{ij}\times RP_{ij}$$

(7) $$RP_{ij}=RP_{zj}*\frac{CDI_{i}}{CDI_{Z}}$$

where DEH is the economic loss related to hospitalization; DEI means the economic loss related to disease; j refers to the type of disease caused by PM_2.5_ pollution; $HE_{ij}$ indicates that the residents in i city suffer from the additional medical expenses incurred as a result of class j diseases; $E_{ij}$ refers to the health impacts of class j diseases in i city; $RP_{ij}$ represents unit disease treatment or hospitalization costs in i city and $RP_{zj}$ represents unit disease treatment or hospitalization costs in z province (due to the availability of data, only the provincial unit economic loss and the per capita disposable income ratio can be used to calculate the unit economic loss of each city); $GDP_{i}$ refers to per capita GDP in i city (GDP means gross domestic product); and $t_{i}$ represents hospital admission time and also expresses time of missed work (the legal working day is 250).

In general, the formula for DED is used to calculate the economic loss caused by premature death and chronic bronchitis, then the DEH is for two types hospitalization (respiratory diseases and cardiovascular diseases), the DEI is for acute bronchitis and asthma. The total economic loss is the sum of the loss form six health outcomes.

## 3. Results and Discussion

### 3.1. Spatial Distribution of PM_2.5_ Pollution and Urbanization

#### 3.1.1. Spatial Distribution Characteristics of PM_2.5_ Concentration

**Figure 1:**
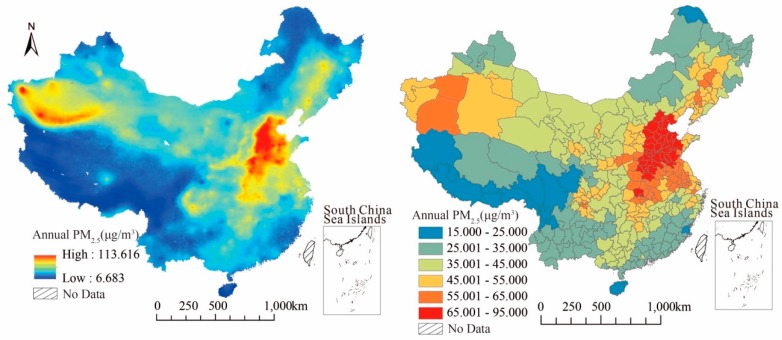
Spatial distribution characteristics of PM_2.5_ concentration in 2015. The left figure is remote sensing of PM_2.5_ concentration and the right figure is PM_2.5_ concentration in city level after using the zonal statistic in ArcGIS (Geographic Information Science).

Figure 1 is the spatial distribution map of PM_2.5_ concentration in China for the year of 2015. The left side is a remote sensing map of PM_2.5_ concentration, while the right side is the annual average of city level that obtained through the zonal statistic in ARCGIS.

First of all, from the annual average of PM_2.5_ concentration, the maximum was 91.378 μg/m^3^ in Hengshui, Hebei province, while the minimum was 15.501 μg/m^3^ in Sanya, Hainan province. The concentration is really different between regions. The number of cities with PM_2.5_ concentration over 35 μg/m^3^ (PM_2.5_ secondary standard from Ambient Air Quality Standard GB3095-2012) was 221, more than two thirds of the total 338 cities. From the perspective of distribution characteristics, PM_2.5_ concentration shows a stepped distribution, where the highest areas were concentrated in the central and eastern regions centered on Beijing–Tianjin–Hebei region, and the medium–high regions were mainly distributed around the highest areas, followed by some cities with high-concentration scattered distribute in the northeast and Xinjiang province. Low-value areas (concentration below 35 μg/m^3^) were mainly located in western China including most of the cities in Tibet, Qinghai, Yunnan, and also in the southeast coastal areas and northeast cities close to the national border. The medium-value areas were mainly distributed in the buffer zone between the high-value and the low-value areas. The PM_2.5_ monitoring points were mainly distributed in urban areas. Compared with the monitoring data, the annual average concentration from remote sensing data was lower, especially in the Beijing–Tianjin–Hebei regions which are the most polluted areas. Although satellite retrieved PM_2.5_ data is also influenced by the appearance of cloud and satellite passing time, it can reflect the average level of regional pollution and is more in line with the actual situation [12,56].

#### 3.1.2. Spatial Distribution Characteristics of Population Density and Urbanization Level

**Figure 2:**
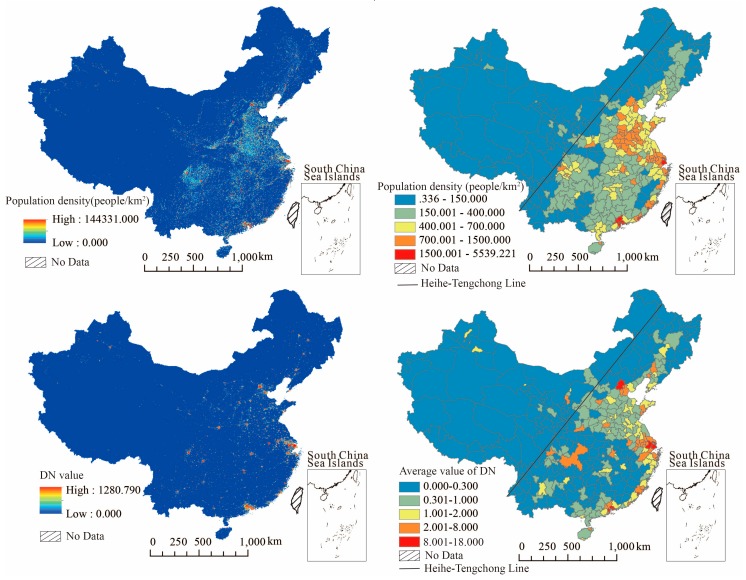
The distribution of population density and urbanization level. The top panel is population density and the bottom panel presents the urbanization level.

In order to clarify the distribution and exposure conditions of populations in various regions, the LanScan data for population density was used to replace the total population at the end of the year (traditional statistical indicators, mainly from the statistical yearbook). For the urbanization level, this study used the nighttime light data instead of the population urbanization rate (traditional statistical indicators, mainly from the statistical yearbook). As shown in Figure 2, the top panel is the spatial distribution of population density, and the bottom panel presents the spatial distribution of urbanization level (DN value).

As shown in Figure 2, the population distribution in east of the Hu Line (also known as the Heihe–Tengchong Line, it is an imaginary line that runs through China and roughly divides the distribution of population into the southeast and northwest) was more intensive, while the population of most cities in the west of the line was sparsely distributed. In particular, the population density of the eastern coastal regions was in the forefront among the whole country; For example the population density of top three cities Shenzhen, Shanghai, and Dongguan was 5539.220 people/km^2^, 3686.905 people/km^2^, and 3522.840 people/km^2^, respectively. Meanwhile population in other areas were more densely distributed in urban agglomerations centered on large cities, such as Beijing, Chengdu, Wuhan, and so on. There was partial overlap in distribution between population density and PM_2.5_ concentration; Thus some areas with dense population distribution were also regions with serious air pollution, especially in the Beijing–Tianjin–Hebei region, Chengdu–Chongqing region and the central plain urban agglomeration. However, compared with the PM_2.5_ concentration distribution, the population distribution did not show a significant stepped distribution. Comparatively, cities with high population density were relatively dispersed and were scattered around large cities in the east of the Hu Line. Therefore, it is necessary to combine the two influencing factors of PM_2.5_ concentration and population density in order to further quantitatively calculate the changes in health risks exposed to polluted air.

From the blow chart of Figure 2 for urbanization level, there were some similar aspects in the distribution characteristics between urbanization level and population density. One of the similar parts was that most of the cities above the national average value were eastern coastal cities and provincial capitals, especially the megacities such as Beijing, Shanghai, Shenzhen, and Guangzhou, where the urbanization levels rank top in the country. Another is that both urbanization level and population density manifested as stepped distribution, that is the value decreases gradually when it spreads outwards from the high-value area. In terms of urbanization, the differences between regions were prominent, with most of the high urbanization areas being scattered in the form of spots, while the high population density areas presented polygon distribution.

#### 3.1.3. Relationship between PM_2.5_ Concentration and Population Density as Well as Urbanization

**Figure 3:**
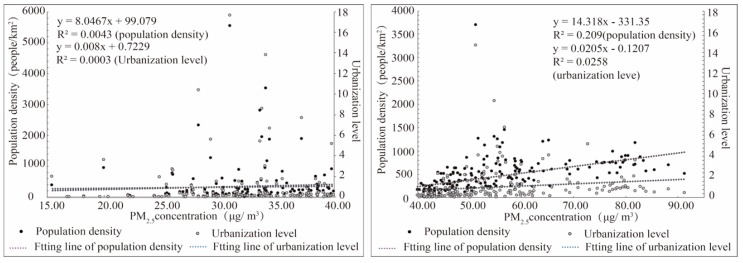
The fitting relationship between PM_2.5_ concentration and population density as well as urbanization level. The (**left**) figure is fitting relationship in low-concentration and the (**right**) one is fitting relationship in high-concentration.

Among the studies exploring the effects of human activities on PM_2.5_ concentrations, population density, and urbanization are two important factors that are indispensable [57,58]. Scholars believe that the continuous promotion of urbanization has produced an agglomeration effect and promoted economic development, but also brought urban diseases such as population agglomeration, traffic congestion, and environmental pollution [58]. In order to analyze the relationship among these three factors, we choose to fit the urbanization level and population density with the PM_2.5_ concentration severally (Figure 3), and the PM_2.5_ concentration has been divided into either low-concentration (left) and high-concentration (right).

On the whole, there was a certain correlation between PM_2.5_ pollution and urbanization level as well as population density. The correlation was not significant in the low-concentration, but in the high-concentration the correlation with population density was more significant, meaning most areas with severe pollution also had a concentrated population distribution. Meanwhile, the cities with high urbanization level were different, some of them were facing the problems of air pollution, but some of cities had good air quality. The reason is that the relationship between urbanization and pollution is extremely complex. Urbanization has brought about population agglomeration, and as a result of population agglomeration and lifestyle change, urban population further expands their demand for basic necessities of life such as food, clothing, housing, and transportation. These changes have further accelerated the development of a correlated industry and the increase of motor vehicles, which has intensified urban air pollution [6]. On the other hand, scholars have put forward different views that under the condition of continuous urbanization, when the social and economic development reaches a higher stage, the effect of urbanization on the environment will be alleviated through technological innovation, structural transformation, and other approaches [59].

### 3.2. Health Risks and Economic Loss of PM_2.5_ Pollution

Compared to the other damage caused by air pollution, the aggravation of air pollution is obviously reflected in health problems [1], and this problem is also the most widespread and publicly concerning topic. Considering the current situation, the air quality has not been improved much after years of control, and the health problems caused by air pollution remain severe [1]. Therefore, we must thoroughly discuss the differences of PM_2.5_ related health problems under different levels of urbanization and make policy makers fully aware of the urgency and danger of environmental problems. Different public health policies need to be proposed according to different characteristics of cities.

#### 3.2.1. Assessment of Health Risks Changes Based on Exposure Response Function

**Figure 4:**
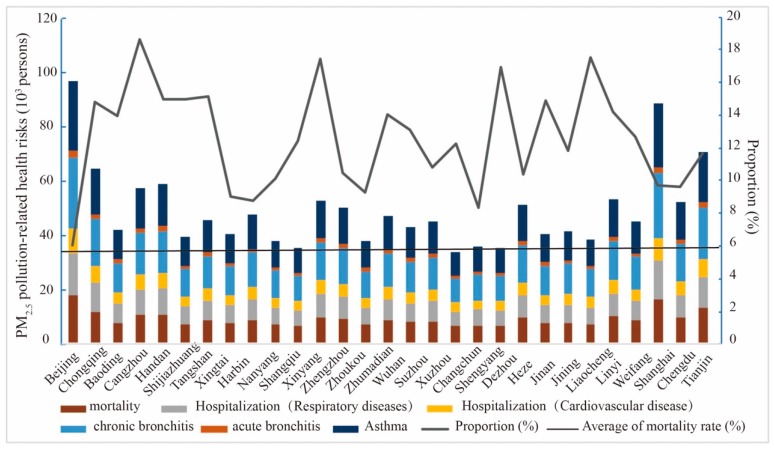
PM_2.5_-related health outcomes change and mortality in the top 30 cities.

The data of PM_2.5_ concentration and population were used to calculate the health risks, including premature death, hospitalization, and disease caused by PM_2.5_ pollution in 338 cities. The estimated results of exposure risk showed that the number of premature deaths, respiratory diseases, and cardiovascular disease due to PM_2.5_ pollution was 0.901 million, 0.806 million, and 0.476 million, respectively. The number of chronic bronchitis, acute bronchitis, and asthma was 1.330 million, 0.138 million, and 1.315 million, respectively. The top 30 cities were selected to draw a histogram. In order to determine the extent of PM_2.5_ impact on health, the proportion of premature deaths caused by PM_2.5_ to the total number of deaths was calculated and plotted a line chart (Figure 4).

As shown in Figure 4, the number of chronic bronchitis and asthma cases caused by PM_2.5_ pollution was the highest, followed by hospitalizations and premature death, while the number of acute bronchitis was small. From the proportion of premature deaths caused by PM_2.5_, compared to 6% of the national average, the proportion of the top 30 cities was higher, indicating that the health risks caused by air pollution were even worse in these cities. From the spatial distribution of the top 30 cities, health risks showed regional differences. The increase in the number of diseases caused by PM_2.5_ pollution mainly occurred in the Beijing–Tianjin–Hebei region and its surrounding areas, Chengdu–Chongqing urban agglomerations, and some provincial capitals such as Jinan, Shenyang, and Wuhan. Different from the distribution of cities with high health risks, cities with a high proportion are mainly located in areas with high PM_2.5_ concentration.

#### 3.2.2. Economic Loss of Health Risks

**Figure 5:**
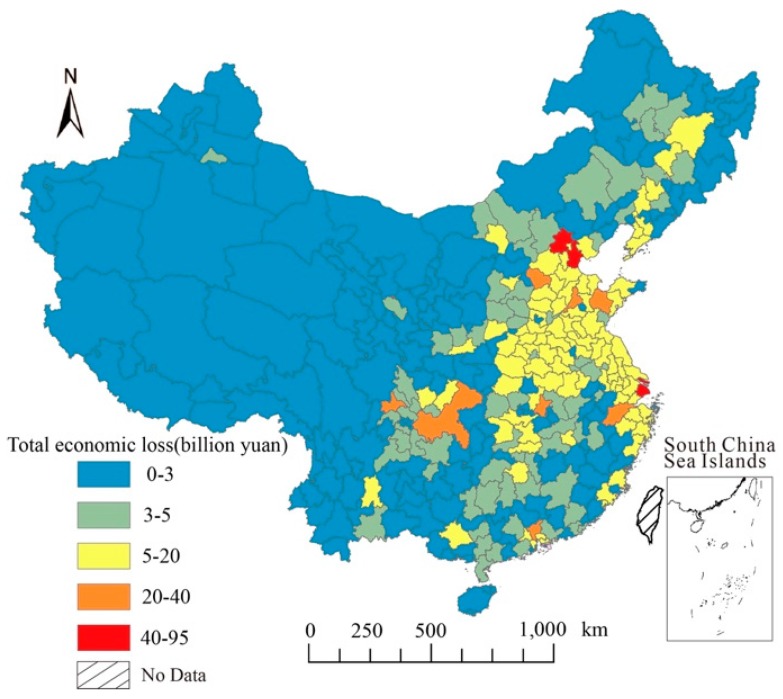
Spatial distribution of total economic loss in 338 Chinese cities.

The health risks caused by air pollution not only bring about an increase in medical costs, but also lead to economic loss in the form of loss of labor and lack of working hours. The economic loss caused by the reduction in working hours and the decline in the supply of labor have reached 0.6%–2.8% GDP [60]. According to the estimation results of health risks and medical related data, the total economic loss of each city can be calculated. The total amount of economic loss was 1.846 trillion yuan, accounting for 2.73% of the total annual GDP. Separately, premature death and chronic bronchitis, which result in permanent or long-term loss of work, had the highest economic loss, with the loss amounting to 1135.732 billion yuan for premature death, while for chronic bronchitis was 670.238 billion yuan. Followed by hospitalization, 5.607 billion yuan for respiratory diseases, cardiovascular diseases was 5.471 billion yuan, as this category of health outcomes require not only medical costs but also the cost of missed work. Others include 0.275 billion yuan for acute bronchitis, and 7.634 billion yuan for asthma. For a more intuitive observation and analysis, the calculation results of the economic loss are shown on the map of China (Figure 5).

From the distribution characteristics, the high-loss areas were mainly distributed in Beijing–Tianjin–Hebei region and surrounding cities, as well as Shanghai, Chongqing, Chengdu, Guangzhou, and other large cities with intensive population distribution and rapid economic development. There were huge differences in economic loss between cities, which means that the loss of high-value areas were hundreds or even thousands of times more than low-value areas. The cities with the top ten economic losses were: Beijing, Shanghai, Tianjin, Chongqing, Shijiazhuang, Weifang, Guangzhou, Wuhan, Chengdu, and Hangzhou (Table 2). These ten cities account for over 20 percent of the total loss and were nearly all distributed in the key area of air pollution in China (The 12th Five Year Plan for Prevention and Control of Atmospheric Pollution in Key Areas has designated 13 regions including Beijing–Tianjin–Hebei, Yangtze River Delta, and Pearl River Delta as the key regions), which also shows that air pollution in these key regions and megacities brings more health damage and economic loss. In other words, more economic loss can be reduced by strengthening air pollution control in these areas.

From Table 2, health outcomes change rank was most similar with economic loss, followed by urbanization level, and finally PM_2.5_ concentration. Based on the ranking of urbanization level, the most similar factor was economic loss, second was health risk, and last was pollution concentration. That means large cities may also face large health outcomes change and high economic loss, for the reasons of the high population density, high medical consumption, large cost of missed work, and so on. Therefore, the exploration of the relationship between urbanization and PM_2.5_ related health loss (whether it is aggravated or reduced), as well as the analysis of the differences in economic loss at different levels of urbanization, must become a new focus in the implementation process of new urbanization and healthy strategies.

#### 3.2.3. Health Risks and Economic Loss under Different Urbanization Levels

**Figure 6:**
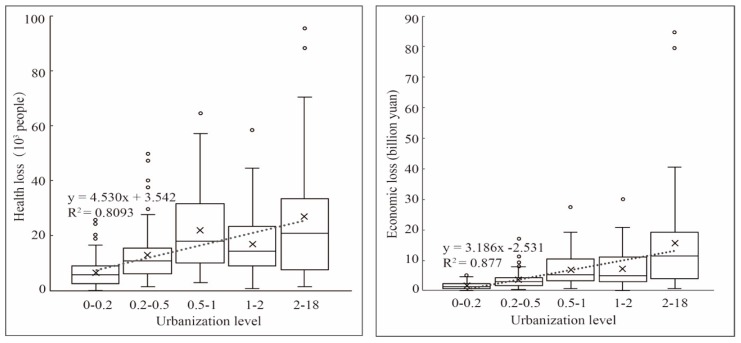
Correlation between urbanization level and PM_2.5_ related health risks as well as economic loss. The (**left**) figure is health risks and the (**right**) one is economic loss.

In order to explore the differences in health problems and economic loss caused by air pollution at different urbanization levels, the urbanization level was divided into five unequal distance stages. The health risks and economic losses were plotted in the box figure and fitted with the urbanization level respectively, as shown in Figure 6. The left side of the figure is health risks and the right side is economic loss).

Comparing Figure 6 with Figure 3, it can be seen that health risks and economic loss have a better fit with urbanization level. R^2^ (goodness of fit) corresponding to air pollution concentration was lower than R^2^ related to health risk and economic loss. The increase of urbanization level had a great impact on the health risks and economic loss, and this effect was more conspicuous than atmospheric pollution. From the left side of Figure 6, with the increase of urbanization level, the average value of health risks was also incremental. The minimum value of each urbanization stage changed little, but as the level of urbanization increased, the change of maximum was getting more remarkable. For economic loss, the changes showed the same tendency as health risks. This stands that in the stage of low urbanization level, due to underdeveloped economy and insignificant agglomeration effect, cities had better environmental quality, lower health risks and economic loss, and smaller difference between cities. In the stage of high urbanization level, the difference of health risk and economic loss between cities was greater, which shows that the related health risks and economic loss had great disparity due to the differences in population density, industrial structures, pollution policy, and geographical environment.

### 3.3. Zoning Control and Policy Recommendations

#### 3.3.1. Regional Classification and Management

**Figure 7:**
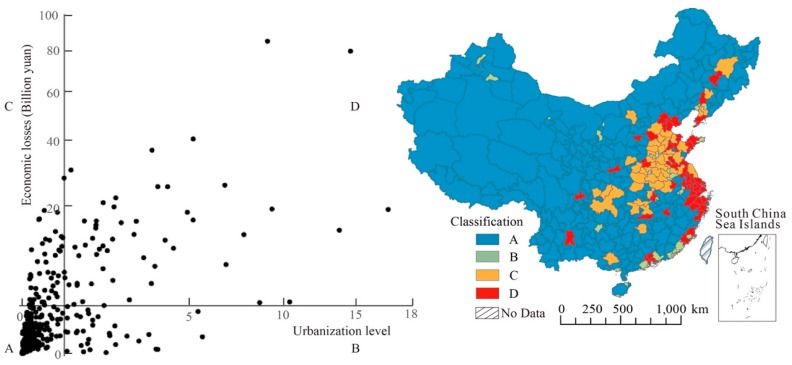
Classification results and distribution of different cities (A is non-key area, B for successful region, C is potential zone, and D represent key area).

To further explore the differences in economic loss under different urbanization levels, taking the urbanization level as the horizontal axis and the total economic loss as the longitudinal axis, the intersection of horizontal and longitudinal coordinates is 1, 5 (corresponding to the classification in Figure 2 and Figure 5), then cities are divided into four categories, and the classification results are visualized, as shown in Figure 7. The III quadrant is type A, which stands for cities with low urbanization and low economic loss; quadrant II corresponds to type B, which are regions with high urbanization but low economic loss, and these cities should be successful examples for others; quadrant IV is Type C, which means cities in this type have a lower urban level but more serious health risks and economic loss, where the population distribution and industrial structures need to be further explored; quadrant I is Type D, which are areas that must be focused on as these areas have a dense population, are exposed to severe polluted environments, and high economic loss due to high medical costs.

From Figure 7, cities of type A accounted for about 62.2% of the total number of cities, and type B, C, and D were 9.3%, 13.1%, and 15.4%, respectively. From the distribution characteristics, the distribution of C and D cities was relatively concentrated, mainly in the eastern and central regions, and cities of type C were mostly distributed around type D. A-Type cities were the most widely distributed, B-type cities were scattered and relatively small in size. Combined with urbanization level and economic loss, it can be seen as follows:

A-type cities were non-key areas with a low level of urbanization and low economic loss. However, in order to avoid further diffusion of C and D agglomerations, the areas around cities of type C and D must pay special attention to the spatial spillover effect of pollution and the transfer of high pollution and high energy consumption industries, so as to avoid the formation of a traditional urbanization mode of developing the economy at the expense of the environment while the level of urbanization is constantly improving.

B-type cities were cities that can be described as “successful models”, such that these were cities with a high level of urbanization but the economic losses are lower. Of course, geographical environment has a great influence, for instance, due to the advantages of better pollution diffusion, some cities in coastal areas such as Xiamen, Zhongshan, and Zhuhai have better air quality. Moreover, the low-concentrations in Zhuhai, Zhongshan, and Xiamen are also due to low emission intensity, especially in the Pearl River Delta region where large numbers of emission control measures have been implemented over this region. For example, Zhongshan and Zhuhai are always influenced by the PM_2.5_ regional transport from Guangzhou; due to regional emission reduction, the concentration in these two cities was low. Furthermore, better air quality brings less health risks and less economic loss. In addition to geographical environment and emission control, there are still some cities have good air quality due to the reasonable industrial structures, a higher threshold for polluting industries, and stronger pollution abatement. B-type cities can become the role model for other cities in the process of urban development.

Among the 338 cities, 53 cities belong to the type D, including 21 provincial capitals and municipalities, such as Beijing, Tianjin, Shanghai, Wuhan, Chengdu, and so on. Type D is the most important type with the greatest need for attention and centralized control. These areas have a high level of urbanization, with a centralized mode of production and lifestyle leading to more serious environmental pollution and health risks. The economic loss was far higher than the national average for the high cost of health care and missed work. Economic loss in the top ten cities accounted for 22.3% of the total national loss, and the urbanization level was higher than the national average, thus air pollution in big cities with a higher urbanization level brings more health risks and economic loss. Taking the economic benefits of environmental governance into consideration, monitoring and managing the environment of these cities can reduce more economic loss.

For these key control areas, firstly, the government should strengthen the monitoring and management of urban pollution then implement emission reduction policies and measures, and the completion of key projects and the operation of monitoring system should be checked. In addition, economic punishment, administrative interviews, and other means should be considered to deal with the illegal emissions. At the same time, due to the high level of urbanization and prominent urban problems, it is particularly necessary to pay attention to the control of motor vehicles and domestic source pollution. In the aspect of public health, the prediction and protection of haze weather are necessary for guiding urban residents to carry out physical protection as an effective way to reduce harm in smog weather. Promoting healthy lifestyles may be necessary and beneficial ways to help people stay healthy. In terms of health care, the government must dedicate access high quality healthcare for related diseases such as bronchitis and asthma, while minimizing the cost of treatment for these diseases through revising medical insurance policy.

Cities belonging to type C were mostly located in the surrounding area of type D, where their own economies are underdeveloped and their urbanization levels are low, but the pollution situation is not optimistic. The economic loss was lower than most of the D-type cities, but was higher than the national average. These regions have the potential to change into type D, because some cities are close to large cities with high level of urbanization, thus receiving high-pollution, energy-intensive industries which have been phased out by many large cities [61]. Although the level of urbanization is low, the industrial output value in these cities is really high. Among the 15 C-type cities, 8 of them have the output value of the secondary industry accounting for more than 50% (the proportion of secondary industry output value is from the China City Statistical Yearbook), especially Baoding, Tangshan, Xuzhou, and other famous resource-based cities or heavy industrial cities. The local industries produce large amount of pollutants into the atmosphere during the production process. Another important reason is that the air quality in these cities is affected by the surrounding type D cities due to air mobility, resulting in health problems and economic loss. Finally, it can be seen from the discussion in Section 3.1.2 that population density is not always correlated well with the DN value, especially for some rural areas. Even when the level of urbanization is low, there is still a relatively dense population distribution, whereby high exposure leads to more health problems.

In addition, it should be noted that for C-type cities, the necessary measures including raising the threshold for new enterprises and at the same time, mobilizing the enthusiasm of existing enterprises in pollution control, promoting emission reduction, improving environmental capacity, and resource allocation efficiency, are also really effective measures. The high economic loss provides a rationale for strengthening the public health services to make them more accessible to people, especially those living in the rural and poverty-stricken areas.

#### 3.3.2. Policy Implications

The Chinese government is aiming to further promote urbanization to stimulate domestic demand and economic growth in the coming decades. Against this backdrop, more people will reside in urban settings and the medical expense continues to grow due to the increasing number of patients and rising medical cost, so health costs must be taken into account when making policy. The following strategies for policy makers can be adopt based on the above analysis.

First, more attention needs to be devoted to the health of residents and the quality of urbanization. In the process of rapid urbanization, it is necessary to pay attention to the health loss cost caused by environmental pollution, especially in areas with high urbanization level (type D). The agglomeration of population, capital, and industries, including pollution-intensive industries, inevitably leads to the increase in pollutant emissions and the health loss in urban place. As China is in period of transition, health costs should be considered in the evaluation system of urbanization quality in the current new urbanization construction. At the same time, the construction of healthy cities should be promoted more widely at the urban scale to arouse people’s awareness of environmental protection and health administration.

Second, in the process of urban development, long-term attention, key control, and differentiated management of health loss are needed. The health loss caused by air pollution needs a long-term evaluation and follow-up investigation process. Therefore, all cities need to keep enough attention other than lower their guard. Despite the current low pollution, high environmental quality, and low economic loss in those cities, pollution control still cannot be ignored. Key cities (type D) need to be integrated into the country’s overall air pollution prevention plan and environmental health policy system. Improving the quality of their environment and the condition of residents’ health is the main focus of the new urbanization construction in the future. Of course, zonal control and cross-regional ecological compensation plans for air pollution also should be taken seriously. There should be more discussion on how to integrate health costs into environmental governance costs in different kinds of cities.

## 4. Conclusions

The PM_2.5_ pollution in the whole country was severe in 2015, with more than 65% of cities and 75% of the population exposed to the polluted environment (PM_2.5_ concentration over 35 μg/m^3^). The morbidity of acute bronchitis and asthma caused by PM_2.5_ pollution was the highest, followed by hospitalizations and premature death, while the morbidity of acute bronchitis was relatively less. The total economic loss due to expose to PM_2.5_ pollution in 2015 was 1.846 trillion yuan, accounting for 2.73% of total annual GDP. With the improvement of urbanization level in different cities, the health risks and economic loss caused by PM_2.5_ pollution are increased. Economic loss in the top ten cities accounted for 22.3% of the total loss, indicating that air pollution in large cities with high level of urbanization lead to more health risks and economic loss. The estimation results for economic valuation should guide decisions on the assessment of environmental health policy options.

Combined with urbanization level and economic loss, 338 cities were classified into four types, including A (non-key area), B (successful region), C (potential zone), and D (key area). At present public health policy in D-type cities with higher levels of urbanization, higher health risks, and higher economic loss, needs to focus on reducing medical costs for PM_2.5_-related diseases. In addition, public facilities and green spaces are needed to encourage people to engage in more physical exercise. For type C cities, the most indispensable goal is to strengthen the quality of public health services making them accessible and timely for people in poor areas to get medical treatment. Although type A cities have less health loss at present, they still need to ensure long periods of time monitoring, especially those around type D cities. In the process of urban development, long-term attention, key control, and differentiated management of health loss are needed, and health loss must be incorporated into environmental management costs and urban quality assessment indicators.

Compared with existing studies which focus on urbanization and pollutants, this paper further discusses the relationship between urbanization and health risks as well as economic loss, which makes the damage caused by atmospheric pollution more intuitive and clearer. While taking economic loss as the criterion, the right to life and health of individuals is neglected. In addition, due to the limitations of research depth and data acquisition, when selecting the exposure response coefficient, a fact that exposure response coefficient is different under different urbanization levels is ignored, which may create some bias in the analysis. In a follow-up study we expect to solve this problem and reduce the estimation errors.

## Figures and Tables

**Table 1:** PM_2.5_ pollution relative risk (RR) factor and baseline incidence rate (BIR).

Health Risk	Health Outcomes	RR (10 μg/m^3^, 95% CI)	BIR Per 10^5^	Reference
Mortality	All-cause mortality	1.019 (1.003, 1.081)	711.0	Wang et al. [47]
Hospitalization	Respiratory diseases	1.022 (1.013, 1.032)	550.9	Li et al. [48]
	Cardiovascular diseases	1.013 (1.007, 1.019)	546.0	Maji et al. [12]
Disease	Chronic bronchitis	1.029 (1.014, 1.044)	694.0	Li et al. [48]
	Acute bronchitis	1.01 (1.005, 1.016)	204.5	Zhang et al. [49]
	Asthma	1.021 (1.015, 1.028)	940.0	Maji et al. [12]

CI: Confidence Interval, RR: relative risk, BIR: baseline incidence rate.

**Table 2:** The properties of cities with the top ten economic loss.

City	PM_2.5_ Concentration (μg/m^3^)	Urbanization Level (DN Value)	Health Outcomes Change (People)	Economic Loss (Billion Yuan)
Beijing	65.221	9.062	95,816.420	84.904
Shanghai	51.235	14.615	88,523.473	79.595
Tianjin	72.756	5.1604	70,459.693	40.503
Chongqing	44.663	3.481	96,563.499	36.513
Shijiazhuang	77.086	1.137	58,607.663	30.034
Weifang	65.886	1.253	44,934.157	27.624
Guangzhou	34.442	6.673	32,836.113	25.585
Wuhan	64.222	3.696	46,945.370	25.189
Chengdu	51.681	4.076	51,813.849	25.142
Hangzhou	43.935	2.274	28,526.093	21.167

DN: Digital Number.

## References

[1] Lin R.S., Sung F.C., Huang S.L., Gou Y.L., Gou H.W., Shaw C.K. (2001). Role of urbanization and air pollution in adolescent asthma: A mass screening in Taiwan. J. Formos. Med. Assoc..

[2] Guan Y., Kang L., Wang Y., Zhang N.N., Ju M.T. (2019). Health loss attributed to PM_2.5_ pollution in China’s cities: Economic impact, annual change and reduction potential. J. Clean. Prod..

[3] Liu M., Huang Y., Jin Z., Ma Z., Liu X., Zhang B., Kinney P.L. (2017). The nexus between urbanization and PM_2.5_ related mortality in China. Environ. Pollut..

[4] Li X., Wang C., Zhang G., Xiao L., Dixon J. (2012). Urbanization and human health in China: Spatial features and a systemic perspective. Environ. Sci. Pollut. Res..

[5] Gong P., Liang S., Carlton E.J., Jiang Q., Wu J., Wang L., Remais J.V. (2012). Urbanisation and health in China. Lancet.

[6] Du C.W., Feng K. (2012). Does urbanization cause air pollution? Empirical evidence from emerging economies. Comp. Econ. Soc. Syst..

[7] Galea S., Vlahov D. (2005). Urban health: Evidence, challenges, and directions. Annu. Rev. Public Health.

[8] Rydin Y., Bleahu A., Davies M., Dávila J.D., Friel S., De Grandis G., Lai K.M. (2016). Shaping cities for health: Complexity and the planning of urban environments in the 21st century. Lancet.

[9] Miao J., Wu X. (2014). Urbanization, socioeconomic status and health disparity in China. Health Place.

[10] Eckert S., Kohler S. (2014). Urbanization and health in developing countries: A systematic review. World Health Popul..

[11] Fischer P.H., Marra M., Ameling C.B., Hoek G., Beelen R., Hoogh K.D., Breugelmans O., Kruize H., Janssen N.A.H., Houthuijs D. (2012). Air pollution and mortality in seven million adults: The Dutch Environmental Longitudinal Study (DUELS). Environ. Health Perspect..

[12] Maji K.J., Ye W.F., Arora M., Nagendra S.M.S. (2018). PM_2.5_-related health and economic loss assessment for 338 Chinese cities. Environ. Int..

[13] Li J., Zhu Y., Kelly J.T., Li J., Zhu Y., Kelly J.T., Jang C.J., Wang S., Hanna A., Yu L. (2019). Health benefit assessment of PM_2.5_ reduction in Pearl River Delta region of China using a model-monitor data fusion approach. J. Environ. Manag..

[14] Leem J.H., Kim S.T., Kim H.C. (2015). Public-health impact of outdoor air pollution for 2nd air pollution management policy in Seoul metropolitan area, Korea. Ann. Occup. Environ. Med..

[15] Lu X., Lin C., Li W., Chen Y., Huang Y., Fung J.C., Lau A.K. (2019). Analysis of the adverse health effects of PM_2.5_ from 2001 to 2017 in China and the role of urbanization in aggravating the health burden. Sci. Total Environ..

[16] Graff Zivin J., Neidell M. (2012). The impact of pollution on worker productivity. Am. Econ. Rev..

[17] Hanna R., Oliva P. (2015). The effect of pollution on labor supply: Evidence from a natural experiment in Mexico City. J. Public Econ..

[18] Deryugina T., Heutel G., Miller N.H., Molitor D., Reif J. (2019). The mortality and medical costs of air pollution: Evidence from changes in wind direction. Natl. Bur. Econ. Res..

[19] Pope C.A., Ezzati M., Dockery D.W. (2016). Fine-particulate air pollution and life expectancy in the United States. N. Eng. J. Med..

[20] Pérez L., Sunyer J., Künzli N. (2009). Estimating the health and economic benefits associated with reducing air pollution in the Barcelona metropolitan area (Spain). Gac. Sanit..

[21] Li H.J., Zhou D.Q., Wei Y.J. (2018). An assessment of PM_2.5_-related health risks and associated economic losses in Chinese cities. Environ. Sci..

[22] Crouse D.L., Peters P.A., Hystad P., Brook J.R., Donkelaar A.V., Martin R.V., Villeneuve P.J., Jerrett M., Goldberg M.S., Pope C.A. (2015). Ambient PM_2.5_, O_3_, and NO_2_ exposures and associations with mortality over 16 years of follow-up in the Canadian census health and environment cohort. Environ. Health Perspect..

[23] Fang D., Wang Q., Li H., Yu Y., Lu Y., Qian X. (2016). Mortality effects assessment of ambient PM_2.5_ pollution in the 74 leading cities of China. Sci. Total Environ..

[24] Jaafar H., Razi N.A., Azzeri A., Isahak K., Dahlui M. (2018). A systematic review of financial implications of air pollution on health in Asia. Environ. Sci. Pollut. Res..

[25] Zeng X.G., Xie F., Zong Q. (2015). Behavior selection and willingness to pay of reducing PM_2.5_ health risk: Taking residents in Beijing as an example. China Pop. Res. Environ..

[26] Huang D.S., Zhang S.Q. (2013). Health benefit evaluation for PM_2.5_ pollution control in Beijing-Tianjin-Hebei region of China. China Environ. Sci..

[27] Wang Q., Wang J., He M.Z., Kinney P.L., Li T. (2018). A county-level estimate of PM_2_._5_ related chronic mortality risk in China based on multi-model exposure data. Environ. Int..

[28] Li T., Zhang Y., Wang J., Xu D., Yin Z., Chen H., Kinney P.L. (2016). All-cause mortality risk associated with long-term exposure to ambient PM_2.5_ in China: A cohort study. Lancet Public Health.

[29] Zheng Y., Zhang Q., Liu Y., Geng G., He K. (2018). Estimating ground-level PM_2.5_ concentrations over three megalopolises in China using satellite-derived aerosol optical depth measurements. Atmos. Environ..

[30] Dobson J., Bright E., Coleman P., Durfee R., Worley B. (2000). A global population database for estimating populations at risk. Photogramm. Eng. Remote Sens..

[31] Xie Y., Dai H., Dong H., Hanaoka T., Masui T. (2016). Economic impacts from PM_2.5_ pollution-related health effects in China: A provincial-level analysis. Environ. Sci. Technol..

[32] Van Donkelaar A., Martin R.V., Li C., Burnett R.T. (2019). Regional estimates of chemical composition of fine particulate matter using a combined geoscience-statistical method with information from satellites, models, and monitors. Environ. Sci. Technol..

[33] Han L., Zhou W., Li W., Li L. (2014). Impact of urbanization level on urban air quality: A case of fine particles (PM_2.5_) in Chinese cities. Environ. Pollut..

[34] Zhang Q., Seto K.C. (2011). Mapping urbanization dynamics at regional and global scales using multi-temporal DMSP/OLS nighttime light data. Remote Sens. Environ..

[35] Shao S., Li X., Cao J.H. (1999). Urbanization promotion and haze pollution governance in China. Econ. Res..

[36] Elvidge C.D., Baugh K.E., Dietz J.B., Bland T., Sutton P.C., Kroehl H. (2019). Radiance calibration of DMSP-OLS low-light imaging data of human settlements. Remote Sens. Environ..

[37] Ma T., Zhou C., Pei T., Haynie S., Fan J. (2012). Quantitative estimation of urbanization dynamics using time series of DMSP/OLS nighttime light data: A comparative case study from China’s cities. Remote Sens. Environ..

[38] Zhao M., Cheng W., Zhou C., Li M., Huang K., Wang N. (2018). Assessing spatiotemporal characteristics of urbanization dynamics in southeast Asia using time series of DMSP/OLS nighttime light data. Remote Sens..

[39] Liu Y., Wang Y., Peng J., Du Y., Liu X., Li S., Zhang D. (2015). Correlations between urbanization and vegetation degradation across the world’s metropolises using DMSP/OLS nighttime light data. Remote Sens..

[40] Ma T., Yin Z., Zhou A. (2018). Delineating spatial patterns in human settlements using VIIRS nighttime light data: A watershed-based partition approach. Remote Sens..

[41] Apte J.S., Marshall J.D., Cohen A.J., Brauer M. (2015). Addressing global mortality from ambient PM_2.5_. Environ. Sci. Technol. Lett..

[42] Apte J.S., Brauer M., Cohen A.J., Ezzati M., Pope C.A. (2018). Ambient PM_2.5_ reduces global and regional life expectancy. Environ. Sci. Technol. Lett..

[43] Zheng S., Pozzer A., Cao C.X., Lelieveld J. (2015). Long-term (2001–2012) concentrations of fine particulate matter (PM_2.5_) and the impact on human health in Beijing, China. Atmos. Chem. Phys..

[44] Pope C.A., Burnett R.T., Thun M.J., Calle E.E., Krewski D., Ito K., Thurston G.D. (2002). Lung cancer, cardiopulmonary mortality, and long-term exposure to fine particulate air pollution. JAMA.

[45] Dockery D.W., Pope C.A., Xu X.P., Spengler J.D. (1993). An association between air pollution and mortality in six US cities. N. Engl. J. Med..

[46] Abrahamowicz M., Schopflocher T., Leffondré K., du Berger R., Krewski D. (2003). Flexible modeling of exposure-response relationship between long-term average levels of particulate air pollution and mortality in the American Cancer Society study. J. Toxicol. Environ. Health A.

[47] Wang G.Z., Wu L.Y., Chen J.B., Song Y.X., Chen R.R. (2017). A CGE-based analysis on PM_2.5_-induced health-related economic effect in Beijing. China Environ. Sci..

[48] Li P., Xin J., Wang Y., Wang S., Li G., Pan X., Liu Z., Wang L. (2013). The acute effects of fine particles on respiratory mortality and morbidity in Beijing, 2004–2009. Environ. Sci. Pollut. Res..

[49] Zhang X., Ou X., Yang X., Qi T., Nam K.-M., Zhang D., Zhang X. (2017). Socioeconomic burden of air pollution in China: Province-level analysis based on energy economic model. Energy Econ..

[50] Yang Z., Liu P., Xu X. (2016). Estimation of social value of statistical life using willingness-to-pay method in Nanjing, China. Accid. Anal. Prev..

[51] Hammitt J.K. (2000). Valuing mortality risk: Theory and practice. Environ. Sci. Technol..

[52] Puig-Junoy J., Zamora A.R. (2015). Socio-economic costs of osteoarthritis: A systematic review of cost-of-illness studies. Seminars in Arthritis and Rheumatism.

[53] Aldy J.E., Viscusi W.K. (1991). Adjusting the value of a statistical life for age and cohort effects. Rev. Econ. Stat..

[54] Viscusi W.K., Magat W.A., Huber J. (2008). Pricing environmental health risks: Survey assessments of risk-risk and risk-dollar trade-offs for chronic bronchitis. J. Environ. Econ. Manag..

[55] Cheng Z., Jiang J., Fajardo O., Wang S.X., Hao J.M. (2013). Characteristics and health impacts of particulate matter pollution in China (2001–2011). Atmos. Environ..

[56] Fang C., Wang Z., Xu G. (2016). Spatial-temporal characteristics of PM_2.5_ in China: A city-level perspective analysis. J. Geogr. Sci..

[57] Wang S., Fang C., Guan X., Pang B., Ma H. (2014). Urbanization, energy consumption, and carbon dioxide emissions in China: A panel data analysis of China’s provinces. Appl. Energy.

[58] Lin B., Zhu J. (2014). Changes in urban air quality during urbanization in China. J. Clean. Prod..

[59] Sadorsky P. (2018). The effect of urbanization on CO_2_ emissions in emerging economies. Energy Econ..

[60] Wang D.W. (2007). Changes in the relationship between labor supply and labor demand and china’s economic growth in the low-fertility era. Chin. J. Pop. Sci..

[61] Long R., Shao T., Chen H. (2016). Spatial econometric analysis of China’s province-level industrial carbon productivity and its influencing factors. Appl. Energy.

